# Stem-cell-derived beta cells mature metabolically upon murine engraftment

**DOI:** 10.1007/s00125-025-06474-8

**Published:** 2025-07-02

**Authors:** Eliisa Vähäkangas, Jonna Saarimäki-Vire, Hossam Montaser, Väinö Lithovius, Solja Eurola, Hazem Ibrahim, Emilia Kuuluvainen, Diego Balboa, Pekka Katajisto, Tom Barsby, Timo Otonkoski

**Affiliations:** 1 Stem Cells and Metabolism Research Program, Faculty of Medicine, University of Helsinki, Helsinki, Finland; 2 Molecular and Integrative Biosciences Research Programme, Faculty of Biological and Environmental Science, University of Helsinki, Helsinki, Finland; 3 Institute of Biotechnology, HiLIFE, University of Helsinki, Helsinki, Finland; 4 Department of Cell and Molecular Biology, Karolinska Institutet, Stockholm, Sweden; 5 Department of Pediatrics, Helsinki University Hospital, Helsinki, Finland

**Keywords:** Beta cell, Implantation, Insulin secretion, Metabolism, Mitochondria, Stem cell

## Abstract

**Aims/hypothesis:**

The use of stem-cell-derived islets (SC-islets) as a source for cell-based therapy of type 1 diabetes shows great potential. However, SC-islets have a metabolically immature phenotype compared with primary human islets, the current ‘gold standard’ cell therapy. SC-islet metabolic immaturity is most evident in their aberrant reactivity to pyruvate, which could be associated with clinically significant dysregulation of insulin secretion. We thus aimed to study whether this immature metabolic phenotype persists upon engraftment in mice.

**Methods:**

This study was conducted by differentiating the H1 human embryonic stem-cell line into pancreatic islets (SC-islets) using a well-established seven-stage differentiation protocol. SC-islets were implanted under the kidney capsule of immunocompromised NOD-*scid*-gamma mice for 1–4 months. Metabolic and morphological assays of SC-islets pre- and post-implantation were performed (in parallel to cadaveric donor islets) using LC-MS metabolomics, electron microscopy, immunohistochemistry analyses and dynamic insulin secretion assays.

**Results:**

SC-islets had the capability of dynamically controlling mouse blood glucose levels by 3 months post-implantation. Murine engraftment led to maturation of various metabolic aspects of SC-islets, leading them to resemble human donor islets more closely. Mitochondrial number increased from a mean of 0.38 mitochondria/µm^2^ in vitro to 0.67 mitochondria/µm^2^ following 4 months of engraftment (*p*=0.0004). Conversely, changes in mitochondrial morphology were cell-specific and correlated more with the insulin granule crystallisation status of a given beta cell than the timepoint of the SC-islet sample. Glucose-sensitive tricarboxylic acid cycle activity increased, with the enrichment of labelled carbons into citrate in high glucose increasing from 12% in vitro to 28.7% 4 months post-engraftment (*p*<0.0001). Additionally, glucose-sensitive insulin secretion increased at the same time as pyruvate-reactive insulin secretion decreased, with the pyruvate-to-glucose reactivity ratio decreasing from 2.1 in vitro to 0.5 at 4 months post-engraftment (*p*=0.013). Lowered pyruvate reactivity was accompanied by the downregulation of the pyruvate/lactate transporter monocarboxylate transporter 1 (MCT1).

**Conclusions/interpretation:**

An in vivo environment is beneficial for the metabolic maturation of SC-islets, leading them to more closely resemble human donor islets. We show that the aberrant metabolite trafficking pathways seen in SC-islets are robustly diminished following engraftment. Our results suggest that metabolic dysregulation is not a major safety concern for clinical SC-islet implantation after prolonged periods of engraftment.

**Graphical Abstract:**

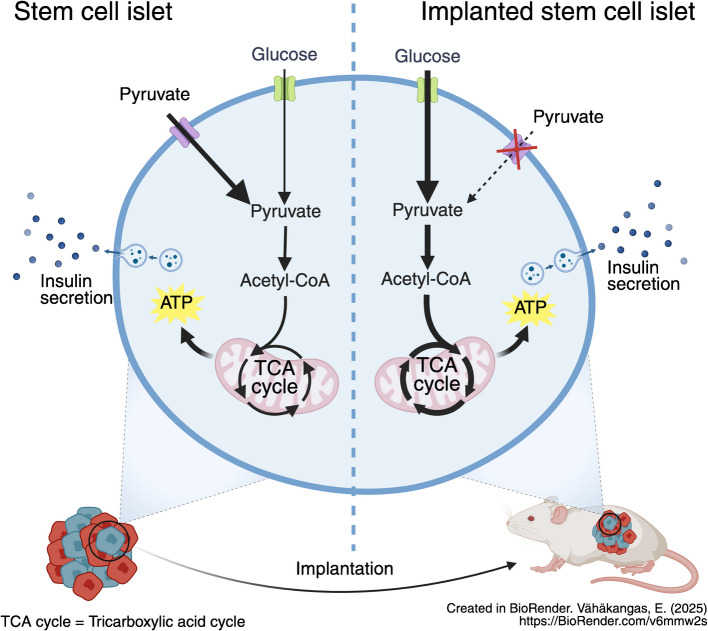

**Supplementary Information:**

The online version contains peer-reviewed but unedited supplementary material available at 10.1007/s00125-025-06474-8.


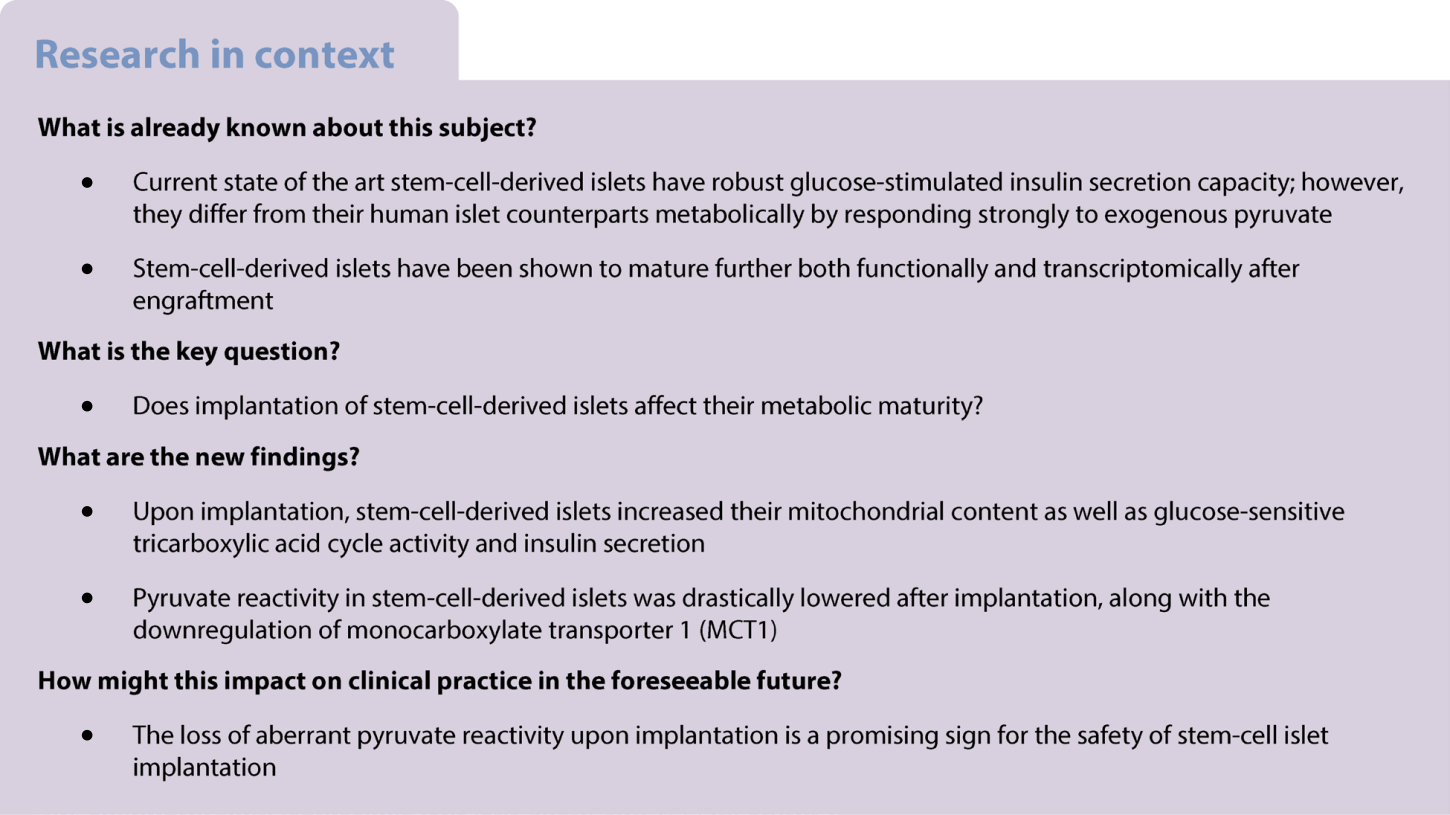


## Introduction

Cell replacement therapy for the treatment of type 1 diabetes, a disease caused by the autoimmune ablation of insulin-secreting beta cells, relies on cadaveric human islets. However, donor islets can be hard to source and successful clinical therapy requires high islet numbers to achieve therapeutic dosages, often necessitating multiple donors for a single recipient [1]. Consequently, functionally mature stem-cell-derived islets (SC-islets) have high therapeutic value as an alternative and renewable source for transplantable cells.

Making highly pure and glucose-responsive SC-islets has become a reality in recent years [2–8]. However, SC-islets still differ from human islets transcriptomically and metabolically [2, 8–10]. An important distinction is the low incorporation of labelled carbons from glucose into tricarboxylic acid (TCA) cycle intermediates in SC-islets, indicative of a lower mitochondrial metabolic coupling to glucose sensing [2]. Furthermore, SC-islets in vitro have higher sensitivity than human islets to non-glucose fuels, most clearly demonstrated for pyruvate [9]. This represents a potential safety concern for SC-islet implantation, as pyruvate released into the bloodstream (along with lactate) upon exercise could induce hypoglycaemia. This is the case in exercise-induced hyperinsulinism, a rare genetic condition caused by the failure to silence monocarboxylate transporter 1 (MCT1), a pyruvate/lactate transporter [11, 12].

As human trials with SC-islet transplants are already ongoing, further understanding of the function and maturity of transplanted SC-islets has immediate translational impact for treatment safety. Interestingly, after murine implantation, SC-islets in vivo spontaneously adopt gene expression changes that are consistent with increased beta cell maturity [2, 10]. However, previous studies have not comprehensively assessed the metabolic maturation of grafted SC-islets.

## Methods

### SC-islet differentiation

The use of human embryonic stem-cell lines approved by the EU Human Pluripotent Stem Cell Registry (hPSC^reg^; https://hpscreg.eu/) for in vitro experimental work does not require a specific ethical approval according to Finnish legislation. The H1 embryonic stem-cell line (mycoplasma negative) (WiCell, WA01) was differentiated to SC-islets with a seven-stage protocol detailed by Barsby et al [13]. Briefly, the cells are induced via specified small-molecule- and growth-factor-supplemented medias to differentiate first through definitive endoderm, primitive gut tube and posterior foregut-like states in monolayer to pancreatic progenitors. The pancreatic progenitors are then dissociated and made into uniform aggregates using AggreWell 400 plates (Stemcell technologies no. 34425). After this, the aggregates are moved to suspension and further differentiated through an endocrine progenitor state to the final pancreatic islet-like clusters. The final SC-islets are then matured for a further 3–4 weeks in the seventh and final stage of the differentiation prior to being implanted.

### In vitro culture of primary adult islets

Primary islets were provided by the Nordic Network for Islet Transplantation (Uppsala University), University of Alberta IsletCore (Canada) and the Center Hospitalier Universitaire de Lille (Lille, France). They were maintained in CMRL 1066 (Corning, 15-110-CVR) supplemented with 10% vol./vol. FBS (Gibco, A5256701), 20 mmol/l HEPES (Gibco, catalogue no. 15630-056), 2 mmol/l Glutmax (LifeTechnologies, 35050038) and 100 U/ml penicillin and 100 μg/ml streptomycin (BioNordika, BN-ECB3001D) on ultralow attachment plates (Corning Costar 6-well Clear Flat Bottom Ultra-Low Attachment 3471). Islet donor characteristics are listed in the human islet checklist found in the electronic supplementary material (ESM).

### Flow cytometry

SC-islets were dissociated with TrypLE (Thermo Fisher Scientific 12563029) for 5–10 min in a 37°C water bath and resuspended in 5% vol./vol. FBS-PBS. Cells were fixed and permeabilised using Cytofix/Cytoperm (BD Biosciences 554714) for 20 min. Fixed cells were incubated overnight at 4°C with primary antibodies (anti-INS-647 1:80, Cell Signaling Technology 9008 and anti-GCG 1:160, Sigma-Aldrich G2654) and secondary antibodies (anti-mouse-488 1:500, Thermo Fisher 21202) for 30 min at room temperature in Perm/Wash buffer (BD Biosciences 554714). The cells were run on FACSCalibur cytometer (BD Biosciences); data were collected with CellQuestPro v.4.0.2 (BD Biosciences) and analysed with FlowJo v.10 (BD Biosciences).

### Implantation of SC-islets

Animal experiments were approved by the Animal Welfare Committee of Southern Finland (ESAVI/47888/2023). NOD-*scid*-gamma (NSG; NOD.Cg-*Prkdc*^*scid*^* Il2rg*^*tm1Wjl*^/SzJ, 0055577; The Jackson Laboratory) mice were housed under a 12 h light–dark cycle with food and water available ad libitum. Approximately 450–750 SC-islets were implanted under the left kidney capsule as described in detail in our previous study [2]. Briefly, the mice were anaesthetised with isoflurane. A small incision was made to the mouse skin and peritoneum, through which the kidney was carefully pulled out and a small nick was made to the apical side of the kidney. A glass wand was used to make a tunnel into which the SC-islets were injected through PE-50 tubing (SDR Scientific, PE9050) with a Hamilton syringe (Hamilton, 81341). The kidney capsule was closed with cauteriser, the peritoneum with stitches and skin with staples. Carprofen (20 mg/kg, s.c.) (Zoetis) and buprenorphine (0.05–0.10 mg/kg, s.c.) (RB Pharmaceuticals) were used as analgesics 30 min before the operation and daily for 2 days after implantation.

### Mouse blood glucose monitoring and IPGTT

Mouse blood samples were collected from the saphenous vein once a month to measure human C-peptide levels from plasma of random-fed mice. IPGTT was performed on fasted mice (4–6 h) 1 month and 4 months after implantation. Mice were injected with 3 g/kg of glucose. Blood glucose measurements and blood sampling were conducted prior to the glucose injection as well as at 15, 30, 60 and 90 min after glucose injection. Human C-peptide from all plasma samples was measured using an ELISA kit (Mercodia, 10-1141-01).

### Immunofluorescence staining and quantifications

The SC-islets were fixed in 4% wt/vol. paraformaldehyde (PFA) for 2 h at room temperature followed by overnight fixation at 4°C. The SC-islets were briefly stained with eosin, then embedded in 2% wt/vol. low-melting agarose (Thermo Scientific, BP1360-100**)** and transferred to paraffin blocks. For graft samples, the graft-containing kidney was retrieved after 1 or 4 months of implantation, fixed in 4% wt/vol. PFA for 48 h at room temperature and processed for paraffin embedding. Antigen retrieval of 5 µm deparaffinised sections was carried out using the Decloaking Chamber (Biocare Medical) in 0.1 mol/l citrate buffer (Thermo Scientific, J63950.AP, pH 6) at 95°C for 12 min for all stainings except for v-maf musculoaponeurotic fbrosarcoma oncogene homologue A (MafA) and MCT1. MafA antigen retrieval was conducted in citrate buffer at 95°C for 20 min. Samples for MCT1 staining were processed in Tris-EDTA buffer (10 mmol/l Tris base [Sigma, T1503], 1 mmol/l EDTA [Sigma, E26290], pH adjusted with 1 mol/l HCl to pH 9) at 95°C for 20 min. Afterwards, sections were blocked with UltraVisionProtein Block (Thermo Fisher Scientific, TA-060-PBQ) for a minimum of 20 min and incubated with primary antibodies diluted in 0.1% vol./vol. Tween in PBS at 4°C overnight, followed by 2 h incubation with the corresponding secondary antibody at room temperature. Details of the antibodies used are listed in ESM Table 1. Stained paraffin sections were imaged using Zeiss Axio Imager 2 with ApoTome.

Image quantifications were performed using CellProfiler software version 4.2.4 (https://cellprofiler.org/previous-releases, Broad Institute) [14]. For all cell-type quantifications, nuclei were used to identify cells and their positivity for specific markers was determined based on positivity of staining around the nuclei. The fluorescence intensity of translocase of outer mitochondrial membrane 20 (TOMM20) was measured in areas around the nuclei of all cells. TOMM20 intensity in insulin-positive cells was normalised in relation to TOMM20 intensity of non-insulin-positive cells of the same sample, with the results depicted as fold change. For quantification of MCT1, masks of E-cadherin and MCT1 were made in insulin-positive areas, followed by a co-localisation analysis of the masks. Results are depicted as a percentage of mask co-localisation per E-cadherin area.

### Transmission electron microscopy

For transmission electron microscopy (TEM), SC-islets, graft pieces or human islets were fixed in 2.5% glutaraldehyde (EM-grade, Electron Microscopy Sciences, 16300), 2 mmol/l CaCl_2_ and 0.1 mol/l sodium cacodylate (NaCac) for 2 h at room temperature. Post-fixation was with 1% osmium-tetroxide for 1 h at room temperature, samples were dehydrated with a graded series of ethanol (70%, 96%, 100%), incubated with transitional solvent acetone and embedded gradually in Epon (TAAB, 812). Afterwards, blocks were cut into 60 nm sections (Leica ultracut UCT) and post-stained with uranyl acetate for 30 min and with lead for 20 s. Imaging was done using a Jeol JEM-1400 Transmission Electron Microscope. Beta cells were identified based on their granules. For each cell, images were taken of the full cell (×800 to ×2000) as well as close-up images of areas with mitochondria (×8000). Insulin granules were counted and categorised manually as diffuse, condensed or crystallised with the help of the Fiji by ImageJ’s (version 2, https://imagej.net/software/fiji/downloads) [15] cell counter tool. Mitochondrial morphology metrics were determined from manually circled mitochondria using the measure tool in Fiji by ImageJ.

### Dynamic insulin secretion assay

Dynamic tests of insulin secretion were carried out using the Biorep Diabetes PERI-4.2 perifusion apparatus with a flowrate of 100 μl/min, sampling every 4 min. Fifty primary islets or 30 SC-islets were used for each channel/technical repeat. For graft samples, mice were euthanised with 70% CO_2_, and grafts were removed from the kidney by pulling the kidney capsule to which the grafts were attached. The graft tissue was then dissected from the kidney capsule under a stereomicroscope. After removing excess fat, the graft tissue was gently teased apart with a scalpel into manageable pieces (smaller than 1 mm in diameter) in KRB containing 3 mmol/l glucose. The pieces were then moved to a 15 ml falcon tube and allowed to settle for a few minutes after which the supernatant fraction was moved to a new tube and spun down at 1000 *g* for 5 min, followed by the plating of these easily dissociated cells/aggregates. The larger pieces were dissociated using Collagenase NB 7D Gentle grade (Nordmark, S1747401) 0.5 U/ml at 37°C for 10 min with frequent shaking of the tube, pipetted vigorously after the incubation and spun down at 1000 *g* for 5 min before plating the pelleted cells/aggregates. The graft-derived cell clusters were grown in SC-islet media (CMRL 1066 [Corning, 15-110-CVR] supplemented with 1 mmol/l *N*-acetylcysteine [Sigma-Aldrich, A9165], 10 nmol/l triiodothyronine (T3) [Sigma-Aldrich, T6397] and 0.5 μmol/l ZM-447439 [Selleckchem, S1103]) on a rotator for 48 h before the dynamic test, with Rock inhibitor supplementation (10 mmol/l) (Y-27632, Selleckchem, S1049) for the first 24 h in culture. The full amount of graft-derived aggregates was apportioned to four channels for the dynamic test. All samples were allowed to equilibrate in KRB (128 mmol/l NaCl, 5 mmol/l KCl, 2.7 mmol/l CaCl_2_, 1.2 mmol/l MgCl_2_, 1 mmol/l Na_2_HPO_4_, 1.2 mmol/l KH_2_PO_4_, 5 mmol/l NaHCO_3_, 10 mmol/l HEPES and 0.1% BSA in dH_2_O) with 3 mmol/l glucose (low glucose) (Sigma-Aldrich, G8769) for 90 min. The sequential sampling conditions were as follows: low glucose (3 mmol/l) 5 samples/20 min; high glucose (17 mmol/l) or high pyruvate (10 mmol/l pyruvate with 3 mmol/l glucose) (Lonza, BE13-115E) 5 samples/20 min; high glucose or high pyruvate and exendin-4 (11 nmol/l) (Anaspec, AS-24463) 3 samples/12 min; low glucose 4 samples/16 min; and KCl (30 mmol/l) in low glucose 2 samples/8 min. Samples from each collected fraction were analysed using insulin ELISA (Mercodia, Sweden, 10-1113-10) and all data are shown as fold change of insulin release relative to the first low-glucose step.

### Metabolomic tracing assay

For metabolite tracing assays, 150–200 SC-islets and 150–200 primary islets were used for each technical replicate of [^13^C_6_]glucose or [^13^C_3_]pyruvate labelling. Grafts were extracted from the kidneys of euthanised mice after 1 or 4 months of engraftment. The graft material in KRB containing 3 mmol/l glucose was then gently teased apart with a scalpel under a dissection microscope into manageable pieces (smaller than 1 mm in diameter) and allocated into eight even-sized samples. Samples were counted into wells of a 12-well tissue culture plate in a volume of 1 ml KRB containing 3 mmol/l unlabelled glucose. Samples were then incubated on a rotator plate at 95 rev/min for 90 min at 37°C and 5% CO_2_ before being transferred to Eppendorf tubes and the basal KRB exchanged for a 0.5 ml volume of KRB containing either ‘low’ or ‘high’ concentrations of [^13^C_6_]glucose (Cambridge Isotope Laboratories, CLM 1396) (3 mmol/l, 17 mmol/l) or [^13^C_3_]pyruvate (Cambridge Isotope Laboratories, CLM 2440) (10 mmol/l). Islets were then incubated for 1 h at 37°C and 5% CO_2_. After incubation, islets were washed in cold PBS before cell lysis and metabolite extraction in 75 μl of lysis buffer (80% acetonitrile in dH_2_O). Islets were lysed with mild trituration before centrifugation at 10,000 *g* for 10 min at 4°C. The supernatant fraction was transferred into Chromacol(03-FISV) MS vials with a 300 μl glass insert (Thermo Fisher) and sealed with Chromacol caps with white pre-split septa (Thermo Fisher). The remaining cell pellet was used for DNA quantification as well as insulin content analysis. Samples with very low or non-detectable insulin content (<500 mU/l, with the content of the average included samples being 20 times higher than this) as well as their counterparts (low glucose/high glucose pair) were excluded from the results due to them not containing a substantial content of our cell type of interest. Samples were analysed on a Thermo Q Exactive Focus Quadrupole Orbitrap mass spectrometer coupled with a Thermo Dionex UltiMate 3000 HPLC system (Thermo Fisher Scientific). The HPLC was equipped with a hydrophilic ZIC-pHILIC column (150×2.1 mm, 5 μm) with a ZIC-pHILIC guard column (20×2.1 mm, 5 μm, Merck Sequant). Five microlitres of each sample was used for each assay. Metabolite separation was achieved by applying a linear gradient of organic solvent (80–35% acetonitrile, 20 mmol/l ammonium bicarbonate) at 0.15 ml/min for 16 min at 45°C. Metabolites were analysed using heated electrospray ionisation (H-ESI) with polarity switching (3400 V for positive, 3000 V for negative) at 280°C, with ion transfer at 300°C. Xcalibur 4.1.31.9 software (Thermo Scientific) was used for LC-MS control. Confirmation of metabolite peak specificity was achieved using commercially available standards (Merck, Cambridge Isotope Laboratories and Santa Cruz Biotechnology). LC-MS data quality was monitored throughout the run with running standard mixes, in-house quality controls and blanks for detecting carry over. Peak integration and metabolite isotopologue identification were accomplished using TraceFinder 4.1 SP2 software (Thermo Scientific). Specificity of labelled peaks and isotopologues were confirmed using cell line controls, blank control samples and non-labelled islet samples pre- and post-incubation. Natural ^13^C abundance was assayed using non-labelled cell samples, and confirmed with correction calculations using IsoCor software (https://bio.tools/IsoCor) for TCA cycle and cycle-derived metabolites [16]. Each metabolite peak area was calculated as a percentage of the total non-labelled (M+0) and labelled metabolite present in the sample (‘% ^13^C labelled of total metabolite’).

## Results

### SC-islets implanted in mice take up to 3 months to humanise blood glucose levels

To study the maturation of SC-islets upon engraftment, we differentiated SC-islets from the H1 human embryonic stem-cell line (ESM Fig. 1a) [2, 13]. Differentiations used for the study had, on average, 38% insulin-positive/glucagon-negative cells and 36% glucagon-positive/insulin-negative cells, as assessed by flow cytometry at week 3–4 of the final stage of the differentiation (ESM Fig. 1b, c). At this time, the SC-islets were implanted into the kidney capsule of immunocompromised mice [2, 17].

**Fig. 1:**
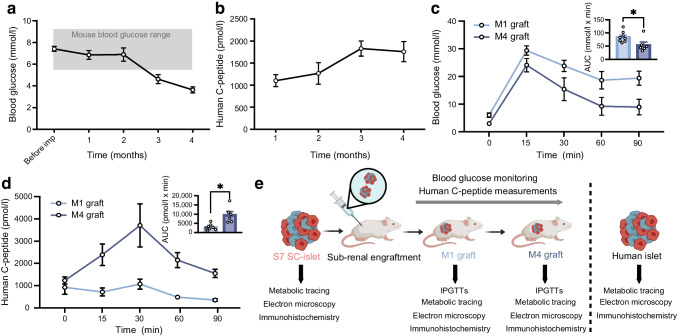
Implanted SC-islets humanise mouse blood glucose after 2 months. (**a**) Blood glucose levels of mice before and after implantation in random-fed mice. Grey box shows ±2 SD of non-transplanted mouse blood glucose levels (before implantation *n*=17, month 1 *n*=15, month 2 *n*=10, month 3 *n*=13, month 4 *n*=9). (**b**) Human C-peptide measured in mouse plasma after implantation in random-fed mice (month 1 *n*=15, month 2 *n*=10, month 3 *n*=12, month 4 *n*=7). (**c**, **d**) Blood glucose (**c**) and human C-peptide (**d**) levels during an IPGTT at 1 month (*n*=8) and 4 months (*n*=6) after implantation. The test was done on fasted mice. (**e**) Schematic depicting the experimental setup of the study, created in BioRender. Vähäkangas, E. (2025) https://BioRender.com/09fqjg6. Data are shown as mean ± SEM. *p≤0.05 (unpaired *t* test with Welch’s correction for AUC)

By 3 months post-implantation, the blood glucose levels of mice implanted with SC-islets reached the human fasting euglycaemic level of below 5.6 mmol/l (reduced from 8 mmol/l) (Fig. 1a) [18]. The lowering of mouse blood glucose levels coincided with increasing levels of circulating human C-peptide (Fig. 1b), confirming the functionality of SC-islet grafts and their connection to the recipient’s vasculature. Graft function was directly assessed by IPGTTs at 1 and 4 months after implantation (Fig. 1c, d). We found that grafts functioned better after 4 months (M4 grafts), as seen by faster glucose clearance, with a significantly lower AUC than after 1 month (M1 grafts) (Fig. 1c). This could be attributed to the higher increase in the levels of human C-peptide secreted during the IPGTT in the M4 grafts (threefold) compared with the M1 grafts (1.2-fold) (Fig. 1d). Total C-peptide secreted during the test was also significantly lower for M1 than for M4 grafts (Fig. 1d). Taken together, our data indicated clear functional maturation of the SC-islet grafts between 1 and 4 months.

### Cell composition changes do not explain increased functionality post-engraftment

**Fig. 2:**
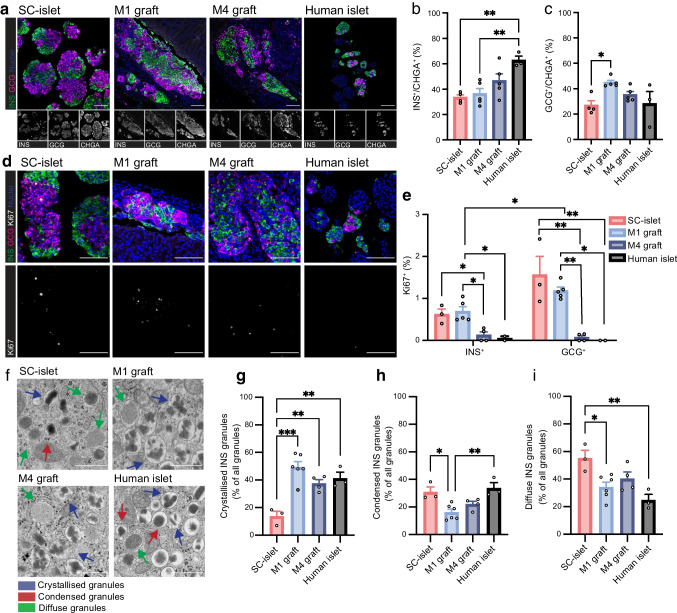
Minor shifts in the composition of endocrine cells seen in implanted SC-islets. (**a**) Representative images of sections immunostained for insulin (green), glucagon (magenta) and CHGA (only shown in single channel image); scale bar, 100 μm. (**b**, **c**) Quantification of insulin- (**b**) and glucagon-positive (**c**) cells as a percentage of the total CHGA^+^ endocrine cell compartment. SC-islet: *n*=4; M1 graft: *n*=5; M4 graft: *n*=5; human islets: *n*=3. (**d**) Representative images of Ki67 stainings (grey) with insulin (green) and glucagon (magenta); scale bar, 100 μm. (**e**) Quantification of the percentage of insulin-positive and glucagon-positive cells with Ki67. SC-islet: *n*=3; M1 graft: *n*=5; M4 graft: *n*=4; human islets: *n*=2. (**f**) Representative TEM images showing insulin granules of different crystallisation status: crystallised (blue arrow); condensed (red arrow); and diffuse (green arrow). Scale bar, 500 nm. (**g**–**i**) Percentages of crystallised (**g**), condensed (**h**) and diffuse (**i**) insulin granules shown as a percentage of all granules, average per biological replicate in SC-islets (*n*=3), M1 graft (*n*=6), M4 grafts (*n*=4) and human islet samples (*n*=3). Data are shown as mean ± SEM. *p≤0.05, **p≤0.01, ***p≤0.001 (one-way ANOVA with Tukey’s multiple comparisons test for all comparisons, except comparison of Ki67% in insulin-positive vs glucagon-positive cells done by paired *t* test for each timepoint separately). GCG, glucagon; INS, insulin

We next sought to understand how metabolism and/or other aspects of SC-islet biology contribute to the noted functional maturation in vivo and in comparison with non-transplanted primary human islets (Fig. 1e). To assess the possible contribution made by changes in cell composition, we investigated the percentage of insulin-positive beta cells as well as glucagon-positive alpha cells from the overall endocrine cell pool positive for chromogranin A (CHGA) from paraffin sections (Fig. 2a). Beta cell percentage increased slightly by M4, reaching the range of 40–60%, as previously reported for human islets [19, 20] (Fig. 2b). Alpha cell proportions were very similar in human islets and SC-islets pre-engraftment (Fig. 2c). The proportion of alpha cells transiently increased at M1 (Fig. 2c). This could be due to the slightly higher proliferation seen in alpha cells compared with beta cells in SC-islets in M1 grafts (Fig. 2d, e). The proliferation marker Ki67 decreased sharply from its already low levels, both in beta cells and alpha cells between 1 month and 4 months post-implantation (Fig. 2d, e). In M4 grafts, only 0.12% of beta cells and 0.07% of alpha cells showed markers of proliferation. Almost no proliferating alpha or beta cells were detected in the human islet samples (Fig. 2e).

To verify that no gross compositional changes occurred in other endocrine cell populations, we also investigated the percentages of somatostatin-positive delta cells, solute carrier family 18 member A1 (SLC18A1)-positive enterochromaffin-like cells, pancreatic polypeptide-positive gamma cells and ghrelin-positive epsilon cells (ESM Fig. 2a–h). We did not observe any changes in any of the populations following engraftment. SC-islet delta cell percentages stayed stable at around 2% of the population, lower than the human islet counterpart (ESM Fig. 2a, b). The proportion of enterochromaffin-like cells remained stable, at approximately 4% after implantation (ESM Fig. 2c, d). Enterochromaffin-like cells are not naturally occurring islet cells but rather a byproduct of the differentiation, seen in many previous studies of SC-islets and thus are not present in human islets [7]. Gamma cell proportions were below 0.5% in all SC-islet samples, lower than the approximately 1% seen in human islets (ESM Fig. 2e, f). Epsilon cell proportions on the other hand were equally low in all samples, including the human islets (ESM Fig. 2g, h).

### Insulin granule morphology matures by 1 month post-engraftment

As only minimal shifts in endocrine cell composition occurred upon engraftment, we next investigated cell intrinsic factors that could be an additional contributor to increased graft functionality. We first assessed insulin granule morphology using TEM to determine whether visible granule maturation occurred upon SC-islet engraftment (Fig. 2f–i). We categorised insulin granules as ‘crystallised’ with clear irregularly shaped dense content, ‘condensed’ with a circular ‘fried egg’ morphology and ‘diffuse’ granules without any distinct condensation (Fig. 2f). Previous studies have shown that in human fetal pancreas, diffuse and condensed granules are present already at week 8–10.5, whereas crystallised insulin granule morphology only appears at week 14 [21, 22]. We found that the percentage of crystallised insulin granules increased by M1 (Fig. 2g). M1-graft beta cells had almost 50% crystallised granules, matching the level of granule maturity seen in M4 grafts and human islets (Fig. 2g). In contrast, crystallised morphology was found in only 14% of pre-engrafted SC-islets, leaving them with a higher proportion of condensed and diffuse insulin granules (Fig. 2g–i). As granule maturation occurred before the large shifts in C-peptide levels and acquisition of dynamic graft functionality (Fig. 1b), it is unlikely to be a key determinant of functional maturity.

### Mitochondrial content increases upon engraftment

**Fig. 3:**
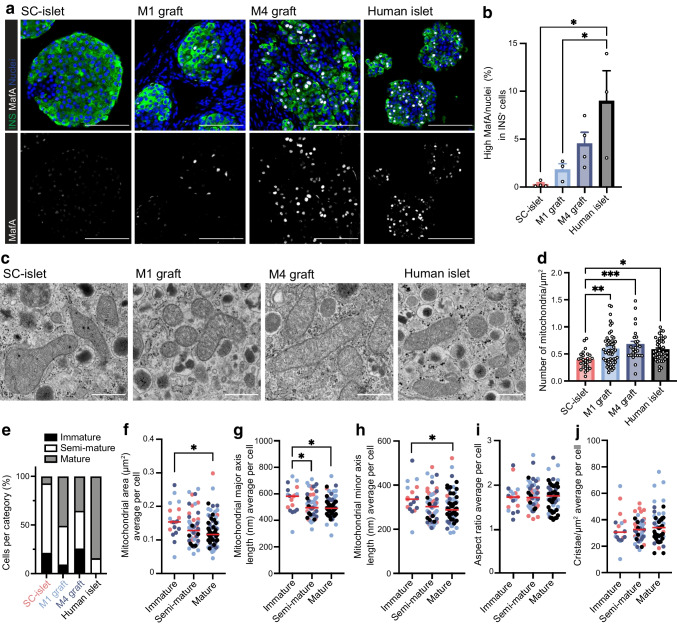
Mitochondrial content increases upon engraftment. (**a**) Representative images of sections stained for MafA (grey) and insulin (green); scale bar, 100 μm. (**b**) Quantification of nuclei with high MafA levels normalised to total nuclei in insulin-positive cells. SC-islet: *n*=4; M1 graft: *n*=3; M4 graft: *n*=4; human islets: *n*=3; mean ± SEM, one-way ANOVA with Tukey’s multiple comparisons test. (**c**) TEM images depicting representative mitochondria from SC-islets, M1 grafts, M4 grafts and human islet samples; scale bar, 500 nm. (**d**) Number of mitochondria per square micrometre quantified from TEM images for cells from SC-islets (pink, 28 cells from 3 biological replicates), M1 grafts (light blue, 55 cells from 6 biological replicates), M4 grafts (blue, 33 cells from 4 biological replicates) and human islets (black, 40 cells from 3 biological replicates); mean with SEM. (**e**) Percentage of cells in each insulin granule crystallisation category. (**f**–**j**) Mitochondrial morphology quantifications from TEM images. Cells from SC-islet (pink, 28 cells from 3 biological replicates), M1 graft (light blue, 55 cells from 6 biological replicates), M4 graft (blue, 33 cells from 4 biological replicates) and human islet (black, 40 cells from 3 biological replicates) categorised into immature (>67% diffuse granules), semi-mature (33–67% diffuse granules) and mature (<33% diffuse granules) insulin granule crystallisation status. Mitochondrial morphology was assessed for area (**f**), major axis (**g**), minor axis (**h**), aspect ratio (minor/major axis ratio) (**i**) and cristae density (cristae per square micrometre) (**j**). Median shown in red. *p≤0.05, **p≤0.01, ***p≤0.001 (one-way ANOVA with Tukey’s multiple comparisons test). INS, insulin

Next, to investigate other possible cell intrinsic maturation mechanisms, we first looked at the protein levels of the beta cell maturation marker MafA, which we have previously reported to be upregulated after engraftment (Fig. 3a) [2]. The proportion of beta cells with high nuclear levels of MafA in SC-islets was very minimal, with an increasing trend following engraftment (Fig. 3b). Human islets had significantly more beta cells with high levels of MafA compared with both SC-islets and M1 grafts (Fig. 3b).

MafA is therefore a good marker of intrinsic beta cell maturation, although it is currently unknown how MafA levels and function directly tie to metabolic maturity. Thus, we next conducted detailed studies of mitochondria, as we have previously reported that mitochondrial oxidative phosphorylation (OXPHOS) genes are upregulated following SC-islet engraftment [2]. We found that mitochondrial content increased gradually in beta cells upon engraftment, as seen by increased mitochondrial number per square micron (0.38 mitochondria/µm^2^ in vitro vs 0.67 at M4, *p*=0.0004), quantified from TEM images, matching the levels of mitochondria seen in human islets (Fig. 3c, d). This mitochondrial increase was corroborated by the increased immunostaining intensity of TOMM20 (a mitochondrial marker protein) in beta (insulin-positive) vs non-beta cell types (ESM Fig. 3a, b). Pre-implantation SC-islet beta cells contained 26% more mitochondria than non-beta cells within the same sample. After implantation, this difference increased gradually, reaching 57% in M4 grafts.

However, the increase in mitochondrial content was not accompanied by clear morphological changes in mitochondria (ESM Fig. 3c–g). Human islets had smaller mitochondria than SC-islets (ESM Fig. 3c). Even the cristae density of the mitochondria stayed similar from one timepoint to the next (ESM Fig. 3g), a parameter often indicative of increased OXPHOS [23], and previously used as a proxy of SC-islet beta maturation [5]. We noted significant heterogeneity between beta cells from the same sample both in their mitochondrial morphology and in their insulin granule maturity.

To address the noted heterogeneity, we devised a way to estimate the degree of beta cell maturity based on their insulin granule crystallisation status, enabling us to compare the mitochondrial morphology between beta cells of differing maturity. Beta cells were categorised into three maturity states based on diffuse granule percentages: mature; semi-mature; and immature. SC-islet beta cells were mainly categorised into the two less-mature states, whereas human islet beta cells primarily fell into the most mature category (Fig. 3e). M1 and M4 graft samples fell between these two. Beta cells with a more-mature insulin granule status had an overall smaller mitochondrial size and area (Fig. 3f–h). The aspect ratio (major axis/minor axis length) of the mitochondria did not dramatically change between the beta cell categories (Fig. 3i). Even with the maturity categorisation there was no significant difference in cristae density (Fig. 3j). Thus, we conclude that mitochondrial morphological parameters correlate poorly with beta cell maturation.

### Utilisation of glucose into TCA-cycle intermediates increases upon engraftment

**Fig. 4:**
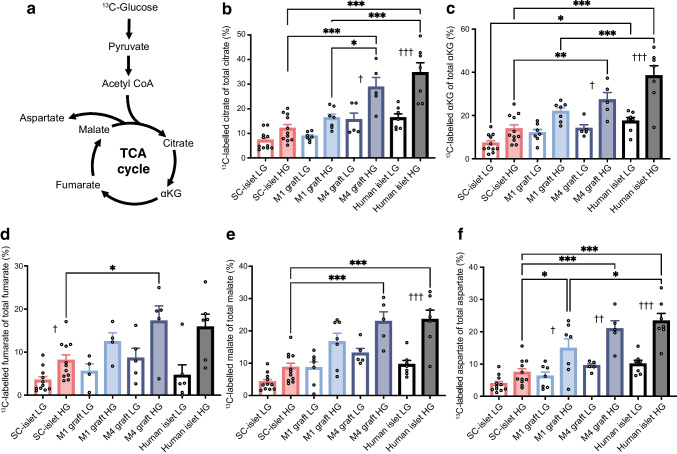
Increased glucose metabolism within mitochondria after engraftment. (**a**) Schematic depicting glucose processing through glycolysis to TCA cycle. Created in BioRender. Vähäkangas, E. (2025) https://BioRender.com/fpywnr2. (**b**–**f**) Percentage of labelled carbons from the total pool of the metabolite, measured in low (3 mmol/l) and high (17 mmol/l) glucose fully labelled with ^13^C, in SC-islets (*n*=11), M1 grafts (*n*=7), M4 grafts (*n*=5) and human islets (*n*=7). Results are depicted for citrate (**b**), αKG (**c**), fumarate (**d**), malate (**e**) and aspartate (**f**). Data are shown as mean ± SEM. *p≤0.05, **p≤0.01, ***p≤0.001 for comparison between sample types; ^†^*p*≤0.05, ^††^*p*≤0.01, ^†††^*p*≤0.001 for low glucose vs high glucose (one-way ANOVA with Tukey’s multiple comparisons test). HG, high glucose; LG, low glucose

To address whether the higher mitochondrial content was connected to increased mitochondrial metabolism, we studied the usage of ^13^C-labelled glucose in basal (3 mmol/l, low glucose) and stimulatory (17 mmol/l, high glucose) glucose concentrations to determine shifts in glucose sensitivity and usage (Fig. 4 and ESM Fig. 4) [9, 13, 24]. Metabolite tracing was conducted in a similar way for SC-islet, pieces of explanted grafts and non-transplanted human islets. The most significant changes were seen in TCA-cycle intermediates in high glucose: citrate increased from 12% in vitro to 28.7% by M4 (*p*<0.0001); fumarate increased from 8% in vitro to 17.2% by M4 (*p*=0.04); α-ketoglutarate (αKG) increased from 13.7% in vitro to 27.2% by M4 (*p*=0.0083); malate increased from 8.6% in vitro to 22.9% by M4 (*p*=0.0002); and aspartate increased from 7.3% in vitro to 20.8% by M4 (*p*<0.0001). All of these showed increased percentages of labelled carbon incorporation following extended engraftment (Fig. 4b–f). The observed increases occurred gradually, achieving human-islet-like glucose incorporation levels at the 4 month timepoint. Glucose utilisation became more concentration-dependent after engraftment, as seen by the relative increase in glucose incorporation under stimulatory glucose concentrations compared with basal levels (Fig. 4b–f).

In addition to core TCA-cycle metabolites, we also detected significant changes in glucose-derived labelling in TCA-derived metabolites (ESM Fig. 4a–d). Such metabolites have been reported as potentially important coupling factors for glucose-induced insulin release [25]. Taken together, carbon usage into the TCA cycle increased post-engraftment, and we therefore next investigated whether this correlated with decreased insulin secretion in response to pyruvate, a fuel with a highly restricted uptake in human islets.

### Pyruvate reactivity decreases upon engraftment

**Fig. 5:**
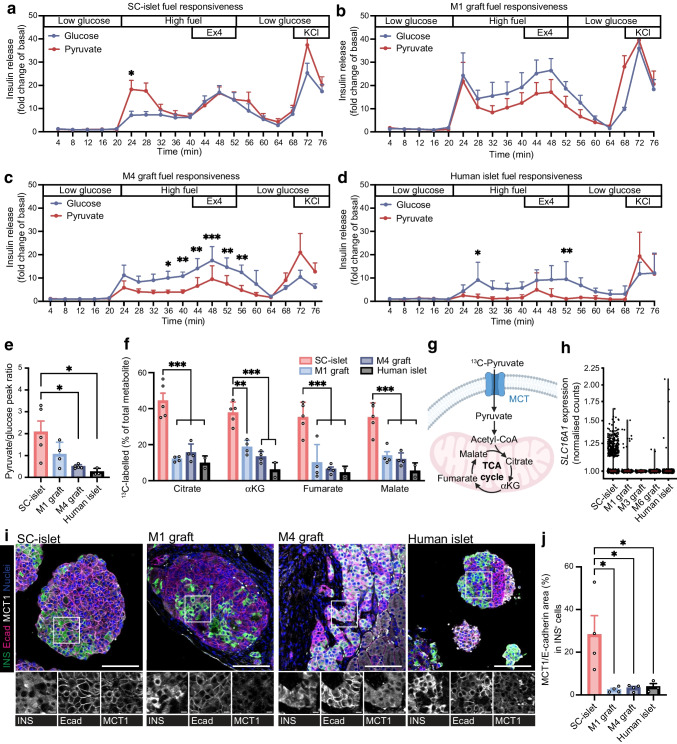
Pyruvate responsiveness lowers after engraftment. (**a**–**d**) Dynamic insulin secretion test run in vitro/ex vivo for SC-islets (**a**, *n*=5), M1 grafts (**b**, *n*=4), M4 grafts (**c**, *n*=5) and human islets (**d**, *n*=3) with either high glucose (17 mmol/l, blue) or high pyruvate (10 mmol/l, red) given at the time indicated as high fuel. Insulin release is shown as fold change from basal secretion. Low glucose =3 mmol/l glucose. Data are shown as mean ± SEM. *p≤0.05, **p≤0.01, ***p≤0.001 (two-way ANOVA with Sidak’s multiple comparison test). (**e**) Ratio of the insulin secretion peak in reaction to high pyruvate (10 mmol/l) vs high glucose (17 mmol/l) from the dynamic insulin secretion test. Data are shown as mean ± SEM. *p≤0.05 (one-way ANOVA with Tukey’s multiple comparisons test). (**f**) Percentage of labelled carbons from the total pool of the metabolite in fully labelled ^13^C[pyruvate] (10 mmol/l) measured in SC-islets (pink, *n*=5), M1 grafts (light blue, *n*=4), M4 grafts (blue, *n*=4) and human islets (black, *n*=2). Data are shown for citrate, αKG, fumarate and malate as mean ± SEM. **p≤0.01, ***p≤0.001 (one-way ANOVA with Tukey’s multiple comparisons test). (**g**) Schematic depicting pyruvate import as well as processing in a cell. Created in BioRender. Vähäkangas, E. (2025) https://BioRender.com/6t4c37x. (**h**) *SLC16A1* (encoding MCT1) expression (normalised to all counts); reanalysis of single-cell RNA sequencing data from Balboa et al [2]. (**i**) Representative images of SC-islet, M1 graft, M4 graft and human islet samples stained for insulin (green), E-cadherin (magenta), MCT1 (grey) and nuclei (blue); scale bar, 100 μm. White box indicates the location of cropped single-channel images shown below; scale bar, 10 μm. (**j**) Quantification of MCT1 co-localisation in E-cadherin-positive cell membrane area in insulin-positive cells. *p≤0.05 (one-way ANOVA with Tukey’s multiple comparisons test). Ecad, E-cadherin; Ex4, exendin 4; INS, insulin

To test glucose- and pyruvate-responsive insulin secretion, we ran dynamic insulin secretion assays in SC-islets and isolated M1 and M4 grafts as well as in donor human islets. All samples showed clear insulin secretion in reaction to KCl-induced cell membrane depolarisation at the end of the test, confirming that they contained viable beta cells throughout the duration of the test (Fig. 5a–d). Although SC-islets responded to glucose, their response to pyruvate was twice as high (Fig. 5a). In M1 grafts, glucose responsiveness was increased, now matching the pyruvate response (Fig. 5b). In M4 grafts, the glucose response was twice as high as the pyruvate response (Fig. 5c), approaching the pattern seen in human islets (Fig. 5d). Exendin-4, a glucagon-like peptide-1 receptor agonist known to potentiate insulin secretion, was effective in all samples in combination with either fuel (Fig. 5a–d). Overall, the relative response of insulin secretion to pyruvate vs glucose decreased sharply upon SC-islet implantation and approached the level seen in human islets (pyruvate-to-glucose reactivity ratio decreased from 2.1 in SC-islets to 0.5 in M4 grafts [*p*=0.013] [Fig. 5e]). Therefore, we conclude that over the course of engraftment, the relative sensitivity to pyruvate was heavily reduced.

To investigate whether the decreased sensitivity to exogenous pyruvate was connected to mitochondrial metabolism, we followed the incorporation of ^13^C-labelled carbons from pyruvate (10 mmol/l) (Fig. 5f). The incorporation of pyruvate-derived carbons to citrate, αKG, fumarate and malate in reaction to pyruvate decreased dramatically by 1 month after engraftment, with similarly low levels seen in M4 grafts and human islets (Fig. 5f).

Pyruvate and lactate are transported into the cell by monocarboxylate transporters [26] (Fig. 5g). Earlier studies have shown that MCT1, encoded by *SLC16A1*, is a disallowed gene in adult beta cells [27]. Its expression leads to dysregulation of insulin secretion [11, 12]. Reanalysis of a previously published single-cell RNA-seq dataset [2] showed that the expression levels of this gene was higher in SC-islets than in graft or human islet samples (Fig. 5h). Immunostaining for MCT1 revealed a cell-membrane-associated signal in SC-islets (Fig. 5i), quantified as co-localisation of MCT1 with the cell membrane protein E-cadherin, encoded by *CDH1* (Fig. 5j). The cell-membrane-associated MCT1 signal was lost after engraftment (Fig. 5i, j). As expected, the MCT1 signal was faint and not distinctly localised to the cell membrane in human islet beta cells (Fig. 5i, j).

## Discussion

SC-islets show great promise as a cell therapy for individuals with type 1 diabetes. However, prior to this study no detailed analysis of the metabolic maturation of SC-islets upon engraftment had been undertaken. This is vital as we have previously shown that SC-islets differ metabolically from human islets in response to non-glucose fuels, especially pyruvate [9]. In this study we showed a sensitivity shift from pyruvate-responsive to glucose-responsive insulin section. This finding is important for ongoing clinical trials using SC-islet cells, as any retention of immature responsiveness to non-glucose fuels could cause dysregulated insulin secretion and hypoglycaemia [11, 12, 28–30].

SC-islets take up to 2 months to humanise mouse blood glucose, clearly a longer time than the few weeks it takes for human islets to achieve this feat [31, 32]. A similar delay in achieving glucose control has been reported in the first clinical trials for SC-islet transplantation [28, 30]. The observed delay could well be explained by the relative metabolic immaturity of SC-islets, which we show in this study to resolve gradually upon implantation.

The metabolic shifts reported in this study were accompanied by a slight increase in beta cell proportion by 4 months. However, this increase is minimal (14%) compared with the large increase seen in circulating human C-peptide (66%). Additionally, our previous in vivo functional imaging has shown that SC-islet grafts composed of pure endocrine cells do not increase in volume, with similar beta cell contribution to endocrine cells as in this study [33]. Thus, we conclude that increased beta cell mass due to endocrine population shifts or graft volume increase is unlikely to be a major player in the increased insulin secretion detected, hence the cause is likely beta cell intrinsic.

One such intrinsic factor could be the increasing mitochondrial content seen in SC-islet beta cells following engraftment. However, we did not detect major alterations in mitochondrial size or morphology (such as cristae density) as reported in other studies using mitochondrial morphology as a proxy for functional maturation in vitro [5]. We conclude that our SC-islets already had a mature mitochondrial morphology in vitro, and the changes recorded are predominantly limited to mitochondrial content. A recent study also reported that the mitochondrial morphology of SC-islets was very similar to that of human islets when an updated differentiation protocol was used [34]. However, opposite to our results, the authors did not find any differences in mitochondrial content between SC-islets and human islet samples, perhaps due to measurements being done on whole aggregates rather than beta cells specifically. Our results indicate that mitochondrial content is a good indicator of beta cell maturity, in line with previous studies [6, 35, 36].

The observed increase in mitochondrial content could occur downstream of MCT1 downregulation. It has been reported that the knockdown or inhibition of MCT1 reduces degradation of peroxisome proliferator-activated receptor γ coactivator 1-α (PGC-1α), which leads to increased mitochondrial content and oxygen consumption [37]. The increased mitochondrial content can in turn lead to a higher TCA-cycle capacity. In addition, the increased incorporation of glucose-derived carbons into TCA-cycle intermediates after implantation could be due to the previously suggested block in late glycolysis being resolved [24, 38].

Identification of the underlying mechanisms for the metabolic maturation occurring after implantation is outside of the scope of the current study. However, certain inferences can be made from our findings and existing data. Time alone is an unlikely explanation, as long-term culture (up to 6 months) in vitro does not lead to the same transcriptomic maturation that occurs after implantation [39], indicating that the change into an in vivo milieu is central for the maturation. Many factors in the in vivo environment could be important. First, fluctuations in nutrient levels have been shown to lead to a shift in mammalian target of rapamycin (mTOR) signalling, mimicking the naturally occurring shift postnatally leading to beta cell maturation [40]. In addition, a multitude of factors in the bloodstream could trigger SC-islet maturation, including circadian cues and systemic hormones such as the thyroid hormone [41–43]. Lastly, direct interactions with other cell types, such as endothelial cells, are known to be important [6]. Taken together, in vivo SC-islet maturation is most likely due to a combination of several environmental factors.

It is reassuring that the immature metabolic features seen in SC-islets in vitro are lost upon implantation into mice. However, care must be taken to interpret these findings in a human setting. Even though possible parallels of in vivo maturation can be inferred to occur in humans, we would recommend ongoing and future clinical trials using SC-islets to verify graft functionality during exercise and/or upon administration of non-glucose metabolites.

In summary, we demonstrate that the previously reported functional and transcriptomic changes occurring upon implantation of SC-islets into mice are accompanied by robust changes in the metabolism of the implanted cells. These metabolic shifts include higher mitochondrial sensitivity to glucose, paralleled with a gradual shutting down of pyruvate-reactive insulin secretion. These results are highly encouraging for future therapies using SC-islets for the treatment of insulin-deficient forms of diabetes.

## Supplementary Information

Below is the link to the electronic supplementary material.

ESM (PDF 9.43 MB)

## Data Availability

All data are available on reasonable request from the corresponding authors.

## Abbreviations

αKG: α-Ketoglutarate
CHGA: Chromogranin A
M1: Graft after 1 month
M4: Graft after 4 months
MCT1: Monocarboxylate transporter 1
MafA: v-maf musculoaponeurotic fibrosarcoma oncogene homologue A
OXPHOS: Oxidative phosphorylation
SC-islets: Stem-cell-derived islets
TCA: Tricarboxylic acid
TEM: Transmission electron microscopy
TOMM20: Translocase of outer mitochondrial membrane 20
