# Supplementary material for: Stem-cell-derived beta cells mature metabolically upon murine engraftment
Source: Diabetologia. 2025 Jul 2;68(9):1997–2010. doi: 10.1007/s00125-025-06474-8 (PMC12361304; doi:10.1007/s00125-025-06474-8)
Supplement: Supplementary file 1 — ESM (PDF 9.43 MB) [file 125_2025_6474_MOESM1_ESM.pdf]

**Electronic supplementary material (ESM) for**

# Stem cell derived beta cells mature metabolically upon murine engraftment

*E. Vähäkangas et al.*

This document includes:

- ESM table 1
- ESM figures 1-4 with legends
- Human islet checklist

**ESM table 1:** Antibodies used for immunofluorescence staining in this study.

| Antibody | Manufacturer | Catalogue number | Dilution |
|---|---|---|---|
| FLEX Polyclonal Guinea Pig Anti-Insulin, Ready-to-Use | Agilent | IR002 | 1:2 |
| Monoclonal Anti-Glucagon antibody produced in mouse | Sigma-aldrich | G2654 | 1:300 |
| Monoclonal Mouse Anti-Human Chromogranin A (Concentrate) | Dako | M0869 | 1:500 |
| Somatostatin Polyclonal Antibody | Invitrogen | PA5-82678 | 1:500 |
| Anti-SLC18A1 antibody produced in rabbit | Sigma-aldrich | HPA063797 | 1:120 |
| Tom20 Antibody (F-10) | Santa cruz | sc-17764 | 1:50 |
| MCT1 Polyclonal antibody | Proteintech | 20139-1-AP | 1:250 |
| Purified mouse anti-E-Cadherin | BD Biosciences | 610182 | 1:250 |
| Anti-MafA antibody [BLR067G] - BSA free | Abcam | ab264418 | 1:250 |
| Goat anti-Rabbit IgG (H+L) Cross-Adsorbed Secondary Antibody, Alexa Fluor™ 647 | Invitrogen | A21244 | 1:500 |
| Goat anti-Guinea Pig IgG (H+L) Highly Cross-Adsorbed Secondary Antibody, Alexa Fluor™ 633 | Invitrogen | A21105 | 1:500 |
| Goat anti-Mouse IgG (H+L) Cross-Adsorbed Secondary Antibody, Alexa Fluor™ 555 | Invitrogen | A21422 | 1:500 |
| Donkey anti-Rabbit IgG (H+L) Highly Cross-Adsorbed Secondary Antibody, Alexa Fluor™ 594 | Invitrogen | A21207 | 1:500 |
| Donkey anti-Rabbit IgG (H+L) Highly Cross-Adsorbed Secondary Antibody, Alexa Fluor™ 488 | Invitrogen | A21206 | 1:500 |
| Goat anti-Guinea Pig IgG (H+L) Highly Cross-Adsorbed Secondary Antibody, Alexa Fluor™ 488 | Invitrogen | A11073 | 1:500 |

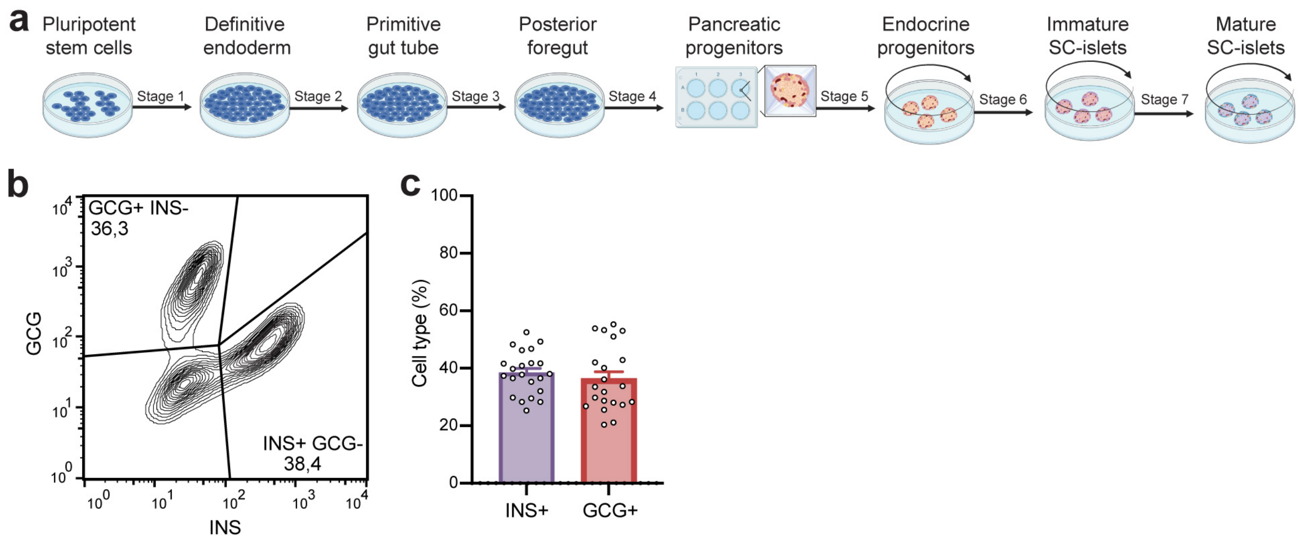


**ESM Figure 1** (a) Schematic depicting the seven-stage differentiation protocol used in the study to obtain SC-islets from pluripotent stem cells, *Created in BioRender. Vähäkangas, E. (2025) https://BioRender.com/ys14bjj*. (b) Representative flow cytometry plot of an SC-islet sample, insulin (INS) on the x-axis and glucagon (GCG) on the y-axis. (c) Percentages of insulin (INS) and glucagon (GCG) positive cells in differentiations used for the study, measured by flow cytometry at week3-4 of the last stage of differentiation. Mean with SEM, n=22.

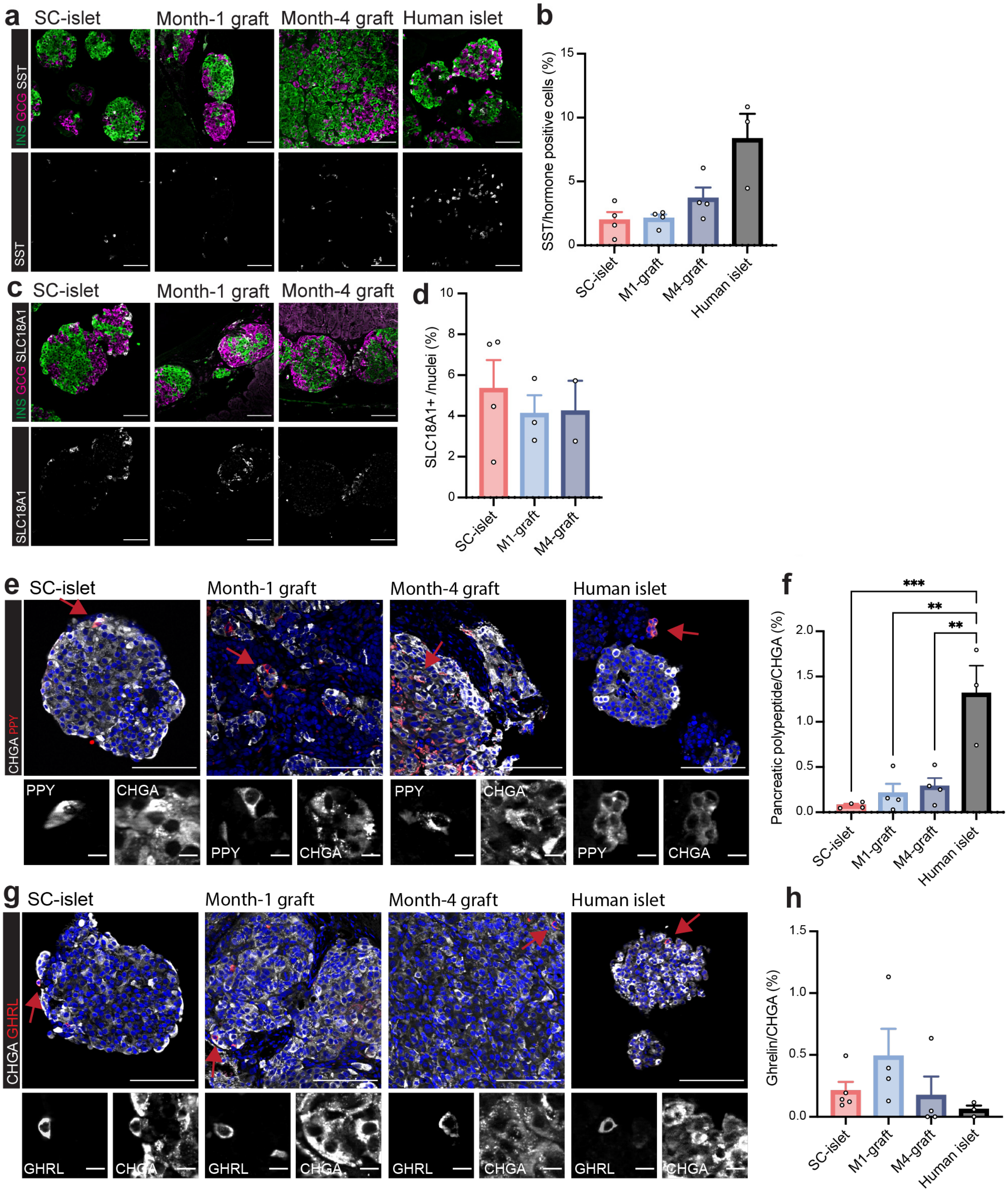


**ESM Figure 2** (a) Representative images of SC-islet, M1-graft, M4-graft and human islet samples stained for Somatostatin (SST, grey), insulin (INS, green) and glucagon (GCG, magenta); scale bar 100µm. (b) Percentage of cells positive for somatostatin (SST) from the total pool of hormone positive, aka insulin, glucagon and somatostatin, positive cells. n=3, except for

human islet samples n=2. (c) Representative images of SC-islet, M1-graft and M4-graft samples stained for SLC18A1 (grey), insulin (INS, green) and glucagon (GCG, magenta). (d) Percentage of SLC18A1 positive enterochromaffin-like cells from all cells (nuclei) for SC-islets (n=4), M1-grafts (n=3) and M4-grafts (n=2). (e) Representative images stained for pancreatic polypeptide (PPY, red) and Chromogranin A (CHGA, grey), scale bar 100μm. Red arrows point out cells which are shown in cropped images of pancreatic polypeptide positive cells; scale bar 10μm. (f) Percentage of cells positive for pancreatic polypeptide (PPY) from the total pool of Chromogranin A (CHGA) positive cells. n=4, except for human islet samples n=3. (g) Representative images stained for ghrelin (GHRL, red) and Chromogranin A (CHGA, grey), scale bar 100μm. Red arrows point out cells which are shown in cropped images of ghrelin positive cells; scale bar 10μm. h) Percentage of cells positive for ghrelin from the total pool of Chromogranin A (CHGA) positive cells. n=4, except for human islet samples n=3. Data is mean with SEM, one-way ANOVA with Tukey's multiple comparisons test., $^{**}P \leq 0.01$; $^{***}P \leq 0.001$.

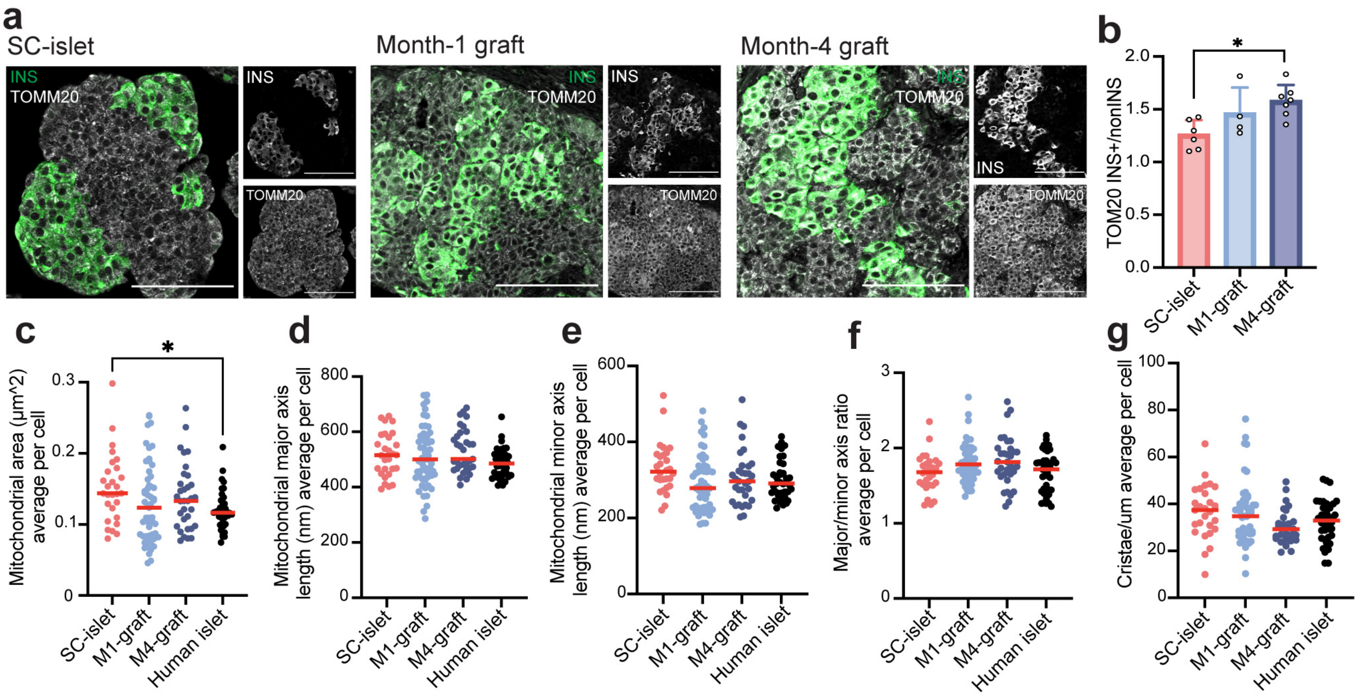


**ESM Figure 3** (a) Representative images of SC-islet, M1-graft and M4-graft samples stained for TOMM20 (grey) and insulin (INS, green); scale bar 100µm. (b) Ratio of TOMM20 staining mean intensity in insulin positive cells compared to non-insulin cells in SC-islets (n=6), M1-grafts (n=4) and M4-grafts (n=7); mean with SEM. (c-g) Mitochondrial morphology measures quantified from TEM images for SC-islets (orange, 28 cells from 3 biological replicates), M1-grafts (teal, 55 cells from 6 biological replicates), M4-grafts (blue, 33 cells from 4 biological replicates) and human islets (black, 40 cells from 3 biological replicates). Median shown as red line. Mitochondrial morphology was assessed for area (c), major axis (d), minor axis (e), aspect ratio (minor/major axis ratio) (f) and cristae density (cristae per square micrometer) (g). One-way ANOVA with Tukey's multiple comparisons test., $*P \leq 0.05$.

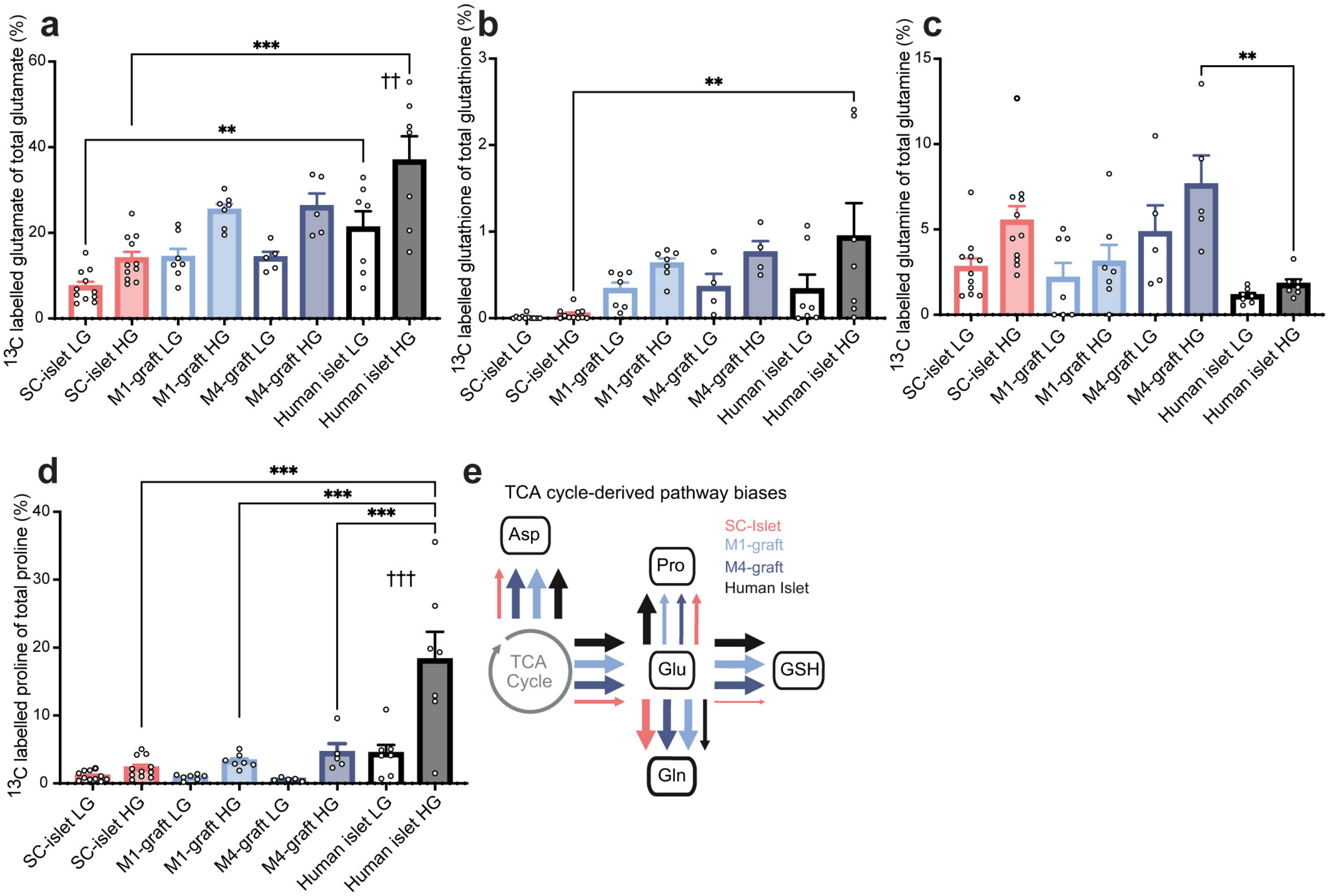


**ESM Figure 4** (a-d) Percentage of labelled carbons from the total pool of the metabolite, measured in low (3mM, LG) and high (17mM, HG) concentrations of fully labelled $^{13}C$-glucose. Measured in SC-islets (n=11), M1-grafts (n=7), M4-grafts (n=5) as well as human islets (n=7). Results depicted for Glutamate (a), Glutathione (b), Glutamine (c) and Proline (d). Data is mean with SEM, one-way ANOVA with Tukey's multiple comparisons test.; Cross symbols indicate internal significance from low to high labelling concentration, asterisk indicates significance between sample types as indicated in figure. ††,**P $\leq$ 0.01; †††,***P $\leq$ 0.001. (e) Schematic depicting the biases of carbon use of SC-islets, M1-grafts, M4-grafts and human islets into TCA-derived metabolites, thickness of arrow indicates level of carbon usage. Metabolites shown are aspartate (Asp), glutamate (Glu), glutamine (Gln), proline (Pro) and glutathione (GSH). *Created in BioRender. Vähäkangas, E. (2025) https://BioRender.com/0fsy2rs*

## Human islet checklist

| Islet preparation | 1 | 2 | 3 | 4 | 5 | 6 | 7 | 8[a] |
|---|---|---|---|---|---|---|---|---|
| **MANDATORY INFORMATION** | | | | | | | | |
| Unique identifier | H2482 | H2489 | H2491 | | | R389 | H2525 | H1183 |
| Donor age (years) | 67 | 65 | 71 | 36 | 61 | 65 | 51 | 46 |
| Donor sex (M/F) | M | M | M | F | F | F | F | F |
| Donor BMI (kg/m$^2$) | 26.1 | 30.1 | 22.4 | 18.9 | 24.7 | 24.4 | 28.1 | 28 |
| Donor $HbA_{1c}$ or other measure of blood glucose control | 38 | 42 | 41 | 32 | 35 | 37 | 35 | 40 |
| Origin/source of islets[b] | Nordic Network for Islet Transplantation, Uppsala | Nordic Network for Islet Transplantation, Uppsala | Nordic Network for Islet Transplantation, Uppsala | Nordic Network for Islet Transplantation, Uppsala | Nordic Network for Islet Transplantation, Uppsala | IsletCore, Alberta | Nordic Network for Islet Transplantation, Uppsala | Center Hospitalier Universitaire de Lille |
| Islet isolation centre | Nordic Network for Islet Transplantation, Uppsala | Nordic Network for Islet Transplantation, Uppsala | Nordic Network for Islet Transplantation, Uppsala | Nordic Network for Islet Transplantation, Uppsala | Nordic Network for Islet Transplantation, Uppsala | IsletCore, Alberta | Nordic Network for Islet Transplantation, Uppsala | Center Hospitalier Universitaire de Lille |
| Donor history of diabetes? Please select | No | No | No | No | No | No | No | No |

| | | | | | | | | |
|---|---|---|---|---|---|---|---|---|
| yes/no from drop down list | | | | | | | | |
| **If Yes, complete the next two lines if this information is available** | | | | | | | | |
| Diabetes duration (years) | | | | | | | | |
| Glucose-lowering therapy at time of death[c] | | | | | | | | |

| **Islet preparation** | **9** | **10** | | | | | | |
|---|---|---|---|---|---|---|---|---|
| **MANDATORY INFORMATION** | | | | | | | | |
| Unique identifier | H1215A | | | | | | | |
| Donor age (years) | 65 | 63 | | | | | | |
| Donor sex (M/F) | | F | | | | | | |
| Donor BMI (kg/m$^2$) | 27.3 | 21,3 | | | | | | |
| Donor $HbA_{1c}$ or other measure of blood glucose control | 37 | 32 | | | | | | |
| Origin/source of islets[b] | Center Hospitalier Universitaire de Lille | IsletCore, Alberta | | | | | | |
| Islet isolation centre | Center Hospitalier Universitaire de Lille | IsletCore, Alberta | | | | | | |
| Donor history of diabetes? Please select yes/no from drop down list | No | No | | | | | | |
| **If Yes, complete the next two lines if this information is available** | | | | | | | | |
| Diabetes duration (years) | | | | | | | | |

| | | | | | | | | |
|---|---|---|---|---|---|---|---|---|
| Glucose-lowering therapy at time of death[c] | | | | | | | | |
